# Test-retest & Inter-rater Reliability of Persian Version of Pediatric Balance Scale in Children with Spastic Cerebral Palsy

**Published:** 2019

**Authors:** Elnaz ALIMIl, Minoo KALANTARI, Ahmad-Reza NAZERI, Alireza AKBARZADE BAGHBAN

**Affiliations:** 1 Department of Occupational Therapy, School of Rehabilitation, Shahid Beheshti University of Medical Sciences, Tehran, Iran; 2 Department of Audiology, School of Rehabilitation, Shahid Beheshti University of Medical Sciences, Tehran, Iran.; 3 Proteomics Research Center, School of Rehabilitation, Shahid Beheshti University of Medical Sciences, Tehran, Iran

**Keywords:** Cerebral palsy, Functional balance, Inter-rater reliability, Test-retest reliability, Pediatric balance scale (PBS)

## Abstract

**Objectives:**

Children affected with spastic cerebral palsy suffering a lot of movement and balance difficulties. Balance is one of the essential variables of movement, which facilitates functional skills. The main purpose of this study was inter-rater & test-retest reliability of Pediatric Balance Scale (PBS) in children with spastic cerebral palsy.

**Materials & Methods:**

In this analytical-descriptive research performed in the rehabilitation centers, south of Tehran, Iran in 2016, psychometric method was used. For investigating the inter-rater reliability, two examiners performed the scale simultaneously with 50 children with spastic cerebral palsy. Moreover, to investigate the test-retest reliability, the scale was implemented by one examiner, in two different sessions, among 50 children with spastic cerebral palsy. There was a two-week period between the first and the second session.

**Results:**

The inter-rater reliability (ICC=0.99), as well as the test-retest reliability (ICC=0.99), was quite high. Standard Error of Measurement (SEM) was acceptable for either test-retest or inter-rater reliability.

**Conclusion:**

PBS is appropriate for measuring functional balance in children with spastic cerebral palsy with mild to moderate motor impairment.

## Introduction

Cerebral palsy is non-progressive brain damage arises before, in the process of, or after birth (1). Cerebral palsy is one of the factors causing disability in childhood, which impacts bodily posture as well as movement and leads to child's restriction of activity and participation (2). This restriction is the product of non-progressive disorder that occurs in the brain. Out of every 1000 successful births, two births result in cerebral palsy (3). Statistics reveal that 70%-80% of cerebral palsy cases are spastic (4). 

Children with spastic cerebral palsy, have diverse difficulties specifically in movement and balance (5). In cerebral palsy, impaired movement is often accompanied by weak balance (6). Balance is the safeguarding of a posture in one specific direction, which is one of the necessary variables of movement. Moreover, balance increases the ability in functional skills (7). Balancing reactions, whether static or dynamic, are weaker in children with cerebral palsy than healthy ones (8). Children with spastic cerebral palsy have abnormal muscle tone as well as postural control. Both of these conditions affect functional balance (9). Functional balance is one of the variables of postural stability that allows the child to safely and independently perform all his/her daily functions in daily life activities and social activities whether at home, school or society in general (10).

 Assessment of balance is one of the main variables for occupational therapists in evaluating school-age children. Occupational therapists should estimate the child's capability for safe and independent functioning in different environments such as home, school, and society (11). Along these lines, they should assess functional balance in order to address whether a spastic child has the required security and independence to fulfill his/her needs in daily tasks (12).

 The test-retest reliability test of the Berg was investigated in children with normal development aged 4 to 13. Reliability was not researched correctly because the needed time period was long for children to complete the static balance items in order to maintain the static posture (11). As a result, due to Berg's burdensome application for children, pediatric balance scale (PBS) was developed as a modified version of Berg (13). The scale of PBS does not require special equipment, performed easily and quickly. This scale is for measuring functional balance of school-age children with mild to moderate motor impairment (12). PBS consists of 14 items in 5 levels and is a criterion reference scale, which assesses the activities that a child should perform at home, school and society in a safe and independent way (14). This test has been translated in Iran and its content validity and face validity have been studied. The content validity index was (CVI=0.97) and the content validity ratio was around 0.80 to 1.00. Moreover, the calculated impact score of face validity was within 2.87 to 4.70 (15). 

The purpose of the study was to the assess inter-rater and test-retest reliability of Persian version of PBS in children with spastic cerebral palsy. 

## Materials & Methods

**Measurers**

Fifty children (27 boys and 23 girls) in the age group of 4-10 yr old with mild to moderate motor impairment (levels 1, 2 and 3 in GMFCS) from the rehabilitation centers in the south of Tehran, Iran were enrolled in Mar 2016 (Table 1). All children were diagnosed with spastic conditions by neurologists. 

Before participating in the research, children's parents were asked to sign the informed consent. This study was approved by the Ethics Committee of the School of Rehabilitation,Shahid Beheshti University of Medical Sciences, Tehran, Iran (approval number:IR.SBMU.RETECH.REC.1395.117) 

In the participating group of children, the spastic cerebral palsy consisted of two types: Hemiplegia and diplegia. Therefore, the level of independency of those children were different and, ranged from fully independent functioning without the need for assistive devices to the use of walkers and KAFO which represented balancing problems. However, as an inclusion criterion, the children had to be able to stand for four seconds without the aid of their hands. Children with less than two years of mental ability who exhibited severe deficiency in attention, disorder in motor development, severe vision and hearing impairment as well as a severe deficiency in language perception, excluded from this research because the presence of such deficiencies would have greatly affected the result of the study. All participants benefited from the occupational therapy services (1 to 4 sessions a week) from the time they had participated in the study (Table 1).

**Table 1:** Characteristics of the 50 children with spastic cerebral palsy in Tehran, Iran in 2016

Variables		Frequency		Frequency (%)
Age (yr)	4-6	23	46	
	6-8	17	34	
	8-10	10	20	
Gender	Girl	23	46	
	Boy	27	54	
GMFCS level	1	12	24	
	2	9	18	
	3	23	46	
Assistive devices	Yes	38	76	
	No	12	24	
Kind of assistive devices	Cane and AFO	7	18	
	KAFO	2	0.53	
	Cane	1	0.26	
	Walker	3	0.79	
	AFO and Walker	8	21	
	KAFO and Walker	12	31	
	AFO and Cane	5	0.13	
Cerebral palsy	Hemipelegia	21	42	
	Diplegia	29	58	
Number of occupational therapy session per week	1 day	9	18	
	2 d	11	22	
	3 d	18	36	
	4 d	12	24	

**Procedure**

**The test-retest reliability**

For the purpose of measuring test-retest reliability of PBS, 50 children with spastic cerebral palsy (GMFCS levels 1, 2, 3) were evaluated in two sessions by a student with master's degree in occupational therapy and two years of experience working with spastic children. The occupational therapist’ responsibilities included explanation of the items to the children, test performance, scoring the test and maintaining security and comfort for the children. Each item was scored on a scale of 0 to 4. In the beginning, each item was requested from the child and then he/she was given only one opportunity to practice the item before the real performance. During the practice, the occupational therapist provided physical help and/or visual or auditory hints, if needed, to make sure that the child totally understands what he/she is supposed to do. On average, each child needed 15 min to completely finish the test. The setting of the test environment was a quiet room with appropriate ambience within The Rehabilitation Centers, and away from physical obstacles. The area of assessment was fixed during the number of evaluations. The second session of testing took place 14 days after the first one. The attendance of family members was optional during the test performance. 

**Inter-rater reliability**

For the inter-rater reliability research, the test was administered twice to each child by two examiners at the same time and in the same place. In the inter-rater reliability study, all 50 children participated in the process. The administration of the testing process was performed by two occupational therapists having master's degree and two years of experience in the area of physical disability in children. During the test, instructions were given by only one examiner. The two examiners were unaware of one another's scoring procedure and each scored the scale based on personal observation. After the performing of each item by the child, both examiners had to record the score and neither of them was permitted to request an item twice from the child for the purpose of changing the score. After execution of the test for each child, five min were spent to record the total score for the child.

**Statistical analysis**

Data were analyzed using by SPSS 18 (Chicago, IL, USA). To obtain test-retest and inter-rater reliability coefficient, the Interclass Correlation Coefficience (ICC) was calculated. ICCs were calculated for total scores. ICC above 0.80, between 0.60 to 0.80 and less than 0.60 indicates the high, moderate, less reliability in sequence (16). The agreement between raters and also test-retest for each individual items of PBS were analyzed regarding the Kappa test. The following is Kappa interoperation: 0.81-0.99 perfect agreement, 0.61-0.8 substantial agreement, 0.41-0.6 moderate agreement, 0.21-0.4 fair agreement and ≤0.20 poor agreement (17). For assessing the reliability, the calculation of Standard Error of Measurement (SEM) and ICC together are suggested, because ICC is a criterion of reliability and represents scale’s capability to make differentiate between participants, while SEM is absolute reliability and quantifies the precise score (18). For measuring absolute reliability, the SEM was calculated. SEM is described as $\mathrm{SD}\sqrt[{2}]{1-\mathrm{ICC}}$. (19).

## Results

**Test-retest reliability**

For the purpose of test-retest reliability regarding the total score of the Persian version of PBS, The correlation coefficient ICC has been calculated. The amount of ICC is 0.99 as the total point for the Persian version of PBS. since ICC is higher than 0.8, PBS reliability exceeds the high (20) (Table 2).

**Inter-rater reliability**

The analysis of correlation coefficient for the purpose of inter-rater reliability regarding the total score of the Persian version of the PBS indicated high reliability (ICC = 0.99) (Table 2).

**SEM**

The calculation of SEM for both test-retest and inter-rater reliability is indicated in table 2 for the total score of Persian version of PBS. Since the amount of SEM was less than 10% of total mean score, SEM was acceptable (21).

**Table 2:** Test-retest, inter-rater reliability ICC and SEM for total score of Persian version of PBS

Reliability	Mean(sd)	ICC	*P*-value	Sem
Inter-rater reliability	Rater 1: 28.82(18.03)	0.99	<0.001	1.80 1.78
	Rater2: 28.74(17.83)			
Test-retest reliability	28.78(17.97)	0.99	<0.001	1.79

**Test-retest and inter-rater reliability study for each Item of pediatric balance scale (PBS)**

The strength of agreement between inter-rater and test-retest analysis in the Persian version of PBS are shown in Table 3. If the amount of kappa coefficient exceeds 0.75, the level of reliability is great. If it is within 0.4 to 0.75, the reliability level is average to good and if it falls below 0.4, the reliability of the scale is weak (20). The kappa coefficient in the Persian version of PBS for test-retest is within 0.92 to 1 with an average of 0.97 and for the inter-rater is between 0.94 and 1 with an average of 0.76.

**Table 3:** Kappa coefficient in test-retest and inter-rater reliability for each item in Persian version of PBS

Item	KappaTest-retest	KappaInter-rater	P-value	Strength ofAgreement
1. Sitting to sanding	1.00	1.00	<0.001	Very good
2.Standing to sitting	1.00	1.00	<0.001	Very good
3.Transfers	1.00	1.00	<0.001	Very good
4.Standing unsupported	0.97	0.94	<0.001	Very good
5.Sitting with back unsupported and feet supported on the ﬂoor	1.00	1.00	<0.001	Very good
6.Standing unsupported with eyes closed	1.00	0.97	<0.001	Very good
7.Standing unsupported with feet together	1.00	0.97	<0.001	Very good
8.Standing unsupported on one foot in the front	0.91	0.94	<0.001	Very good
9.Standing on one leg	0.97	0.88	<0.001	Very good
10.Turning 360 degree	0.97	0.97	<0.001	Very good
11.Turning to look behind left and right shoulders while standing sill	0.94	0.94	<0.001	Very good
12.Pick up object from the ﬂoor from standingposition	1.00	1.00	<0.001	Very good
13.Placing alternate foot on step stool while standing unsupported	0.97	0.97	<0.001	Very good
14.Reaching forward with outstretched arm while sanding	0.94	0.92	<0.001	Very good

## Discussion

Balance disorder is one of the main challenges in children with spastic cerebral palsy, which leads to diminished capability and independency in the activity of daily living (22). PBS is an authentic and specific tool for measuring functional balance in children with cerebral palsy. The purpose of this study was to assess the inter-rater and test-retest reliability and absolute reliability SEM of the Persian version of PBS. In this research, the inter-rater reliability (0.99) and the test-retest reliability (1.00) were quite high for the scale. 

The results of the current study are similar to the results of previous study (11). Although the type of cerebral palsy has not been pointed out in that study, the participants were included children having spastic cerebral palsy with mild to moderate motor impairment. They suggested PBS for evaluation the functional balance in children with mild to moderate motor impairment. Moreover, the inter-rater reliability of (0.997) and test-retest reliability of (0.998) this scale were studied within groups (11). The reliability results were similar to the obtained results of the present study. Moreover, the studies on test-retest reliability (19, 23), and inter-rater reliability (24) among children with spastic cerebral palsy, demonstrated that PBS had a high reliability for the assessment of functional balance in children with spastic cerebral pals The amount of SEM was calculated. SEM for rater number one, rater number two and test-retest was 0.37, 0.43 and 0.61 in sequence (19). This result is approximate with this current study that resulted 1.80, 1.78 and 1.79 for two raters and test-retest reliability. SEM score of below than 10% of the total mean score is acceptable (21). 

The results of this study show that the Persian version of PBS has a high inter-rater and test-retest reliability for measuring functional balance in children with spastic cerebral palsy (GMFCS levels 1, 2, 3). 

**In Conclusion, **PBS can be used with certainty as a reliable clinical tool to evaluate balance in spastic cerebral palsy children. Because PBS has a scoring system with easy application in clinics and it is accomplished in only 15 min, it is recommended that more research is done regarding reliability assessment in other areas of motor impairment. Since inter-rater reliability study must take place in two sessions, it was difficult to arrange the second session exactly two weeks after the first session for some of the children and their families. Therefore, the required test day was changed to one or two days later or sooner for some of the participating children.
